# Supplementary material for: Catalytic production of impurity-free V3.5+ electrolyte for vanadium redox flow batteries
Source: Nat Commun. 2019 Sep 27;10:4412. doi: 10.1038/s41467-019-12363-7 (PMC6764956; doi:10.1038/s41467-019-12363-7)
Supplement: Supplementary file 1 — Supplementary Information [file 41467_2019_12363_MOESM1_ESM.pdf]

Supplementary Information for

Catalytic production of impurity-free $V^{3.5+}$ electrolyte for vanadium redox flow batteries

Heo et al.

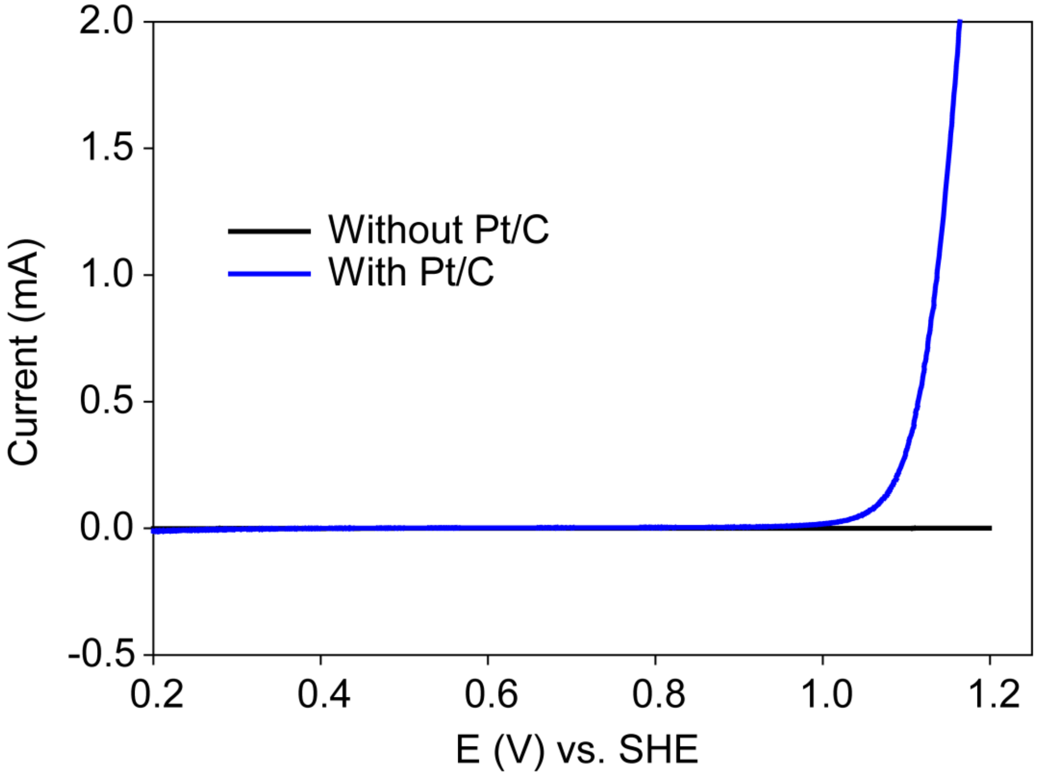

Supplementary Figure 1 Linear sweep voltammetry (LSV) curves for the oxidation of oxalic acid with glass carbon electrode and Pt/C-decorated glassy carbon electrode

To check the catalytic effect of Pt/C on the oxidation of oxalic acid, LSV test was conducted at a scan rate of 0.1 mV s^{-1} with the pristine glassy carbon electrode or the Pt/C-deposited carbon electrode.

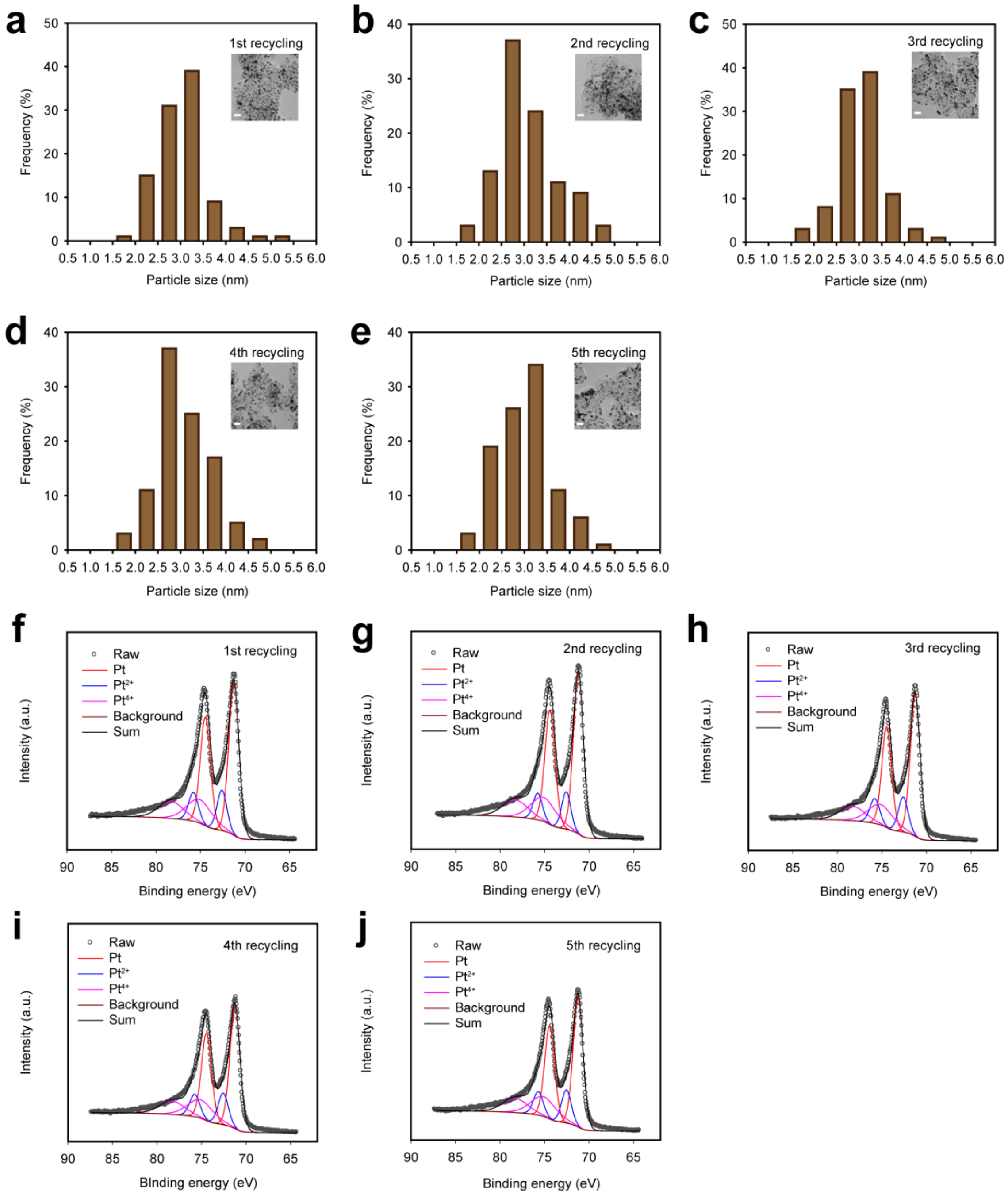

Supplementary Figure 2 Post-mortem analysis of the Pt/C after the repeated catalytic reaction (reduction of V^{4+} to $V^{3.5+}$ with formic acid at 80 °C for 30 min) at different repetition time

Transmission electron microscopy (TEM) images and particle size distributions of the Pt/C catalyst at repetition time of (a) one, (b) two, (c) three, (d) four, and (e) five. Pt 4f X-ray photoelectron spectroscopy (XPS) of the Pt/C catalyst at repetition time of (f) one, (g) two, (h) three, (i) four, and (j) five. Scale bars, 10μm.

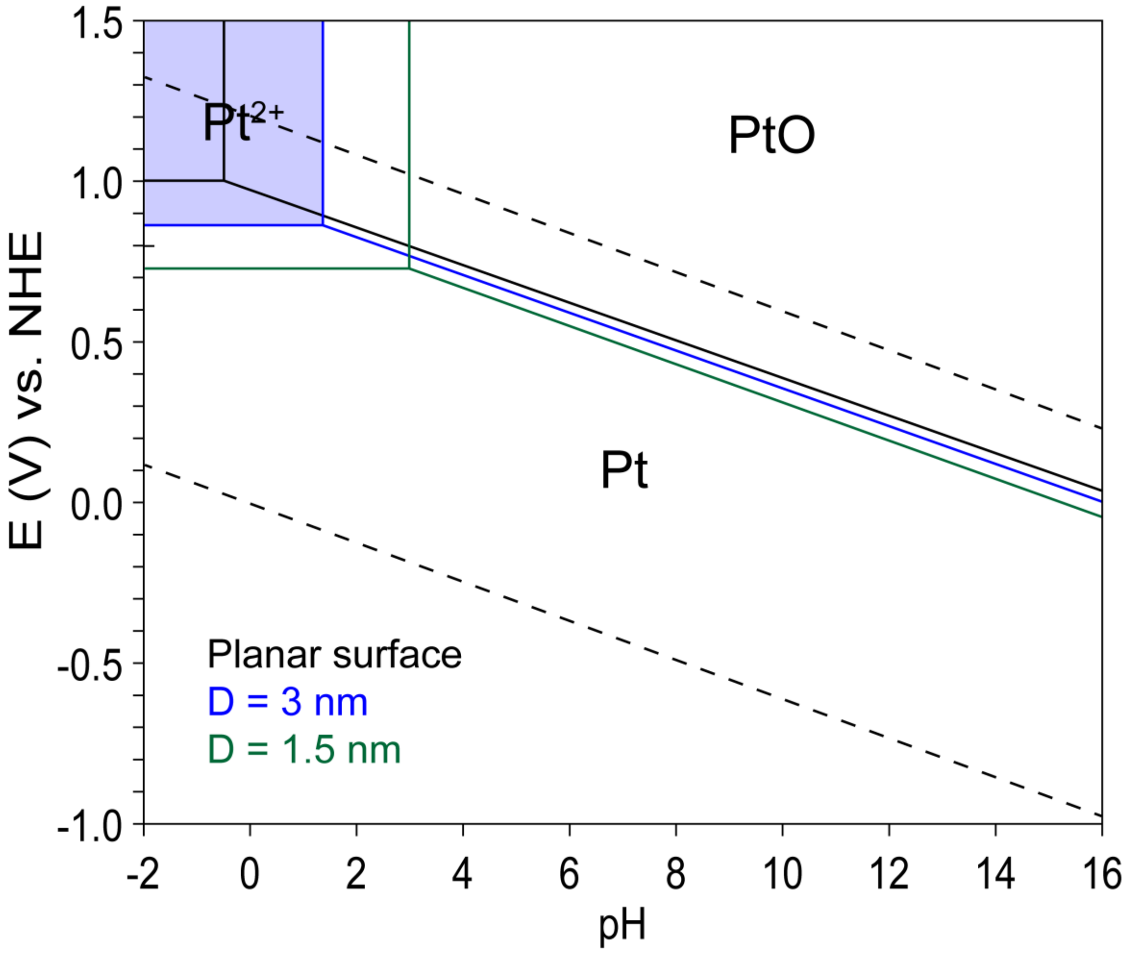

Supplementary Figure 3 Particle-size-dependent potential-pH diagram for Pt / Pt^{2+} ($[Pt^{2+}]$ = 10^{-6} mol dm^{-3})

The highlighted blue area represents the region of Pt^{2+} stability for 3 nm diameter Pt nanoparticle[1]. "Reprinted with permission from (*J. Am. Chem. Soc.* **132**, 11722-11726 (2010)). Copyright (2010) American Chemical Society."

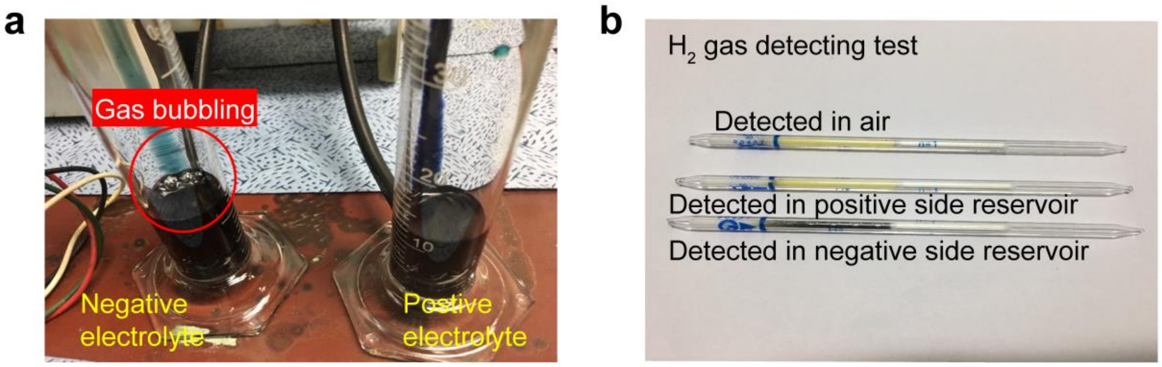

Supplementary Figure 4 Hydrogen evolution reaction (HER) at the negative electrode for the electrolyte prepared with PtRu/C

(a) Optical image showing the hydrogen bubble from the negative electrode. (b) H_2 detecting probes positioned at ambient air, positive side reservoir, and negative side reservoir. The color change from yellow to black indicates the presence of hydrogen gas.

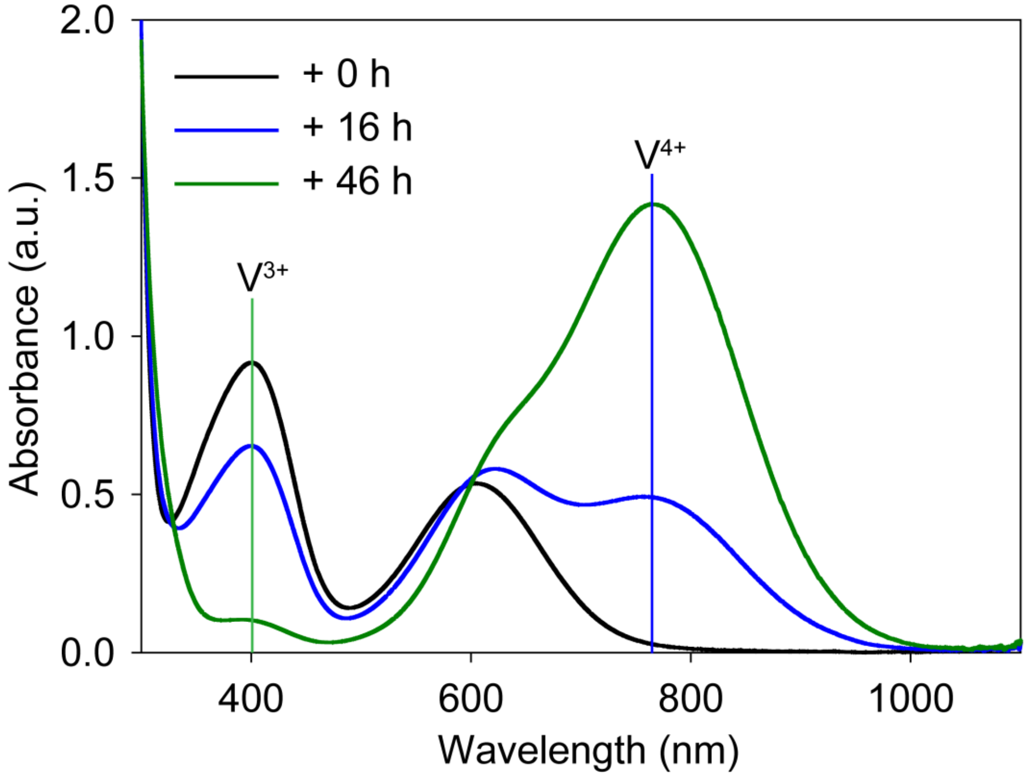

Supplementary Figure 5 Change in the ultraviolet-visible (UV-Vis) spectra of the V^{3+} electrolyte containing Pt/C with storage under ambient air

Since Pt/C accelerates the reduction of oxygen, the V^{3+} produced by the catalytic reduction of V^{4+} can be oxidized to V^{4+}. Therefore, it is checked whether V^{3+} is oxidized to V^{4+} in the presence of Pt/C by monitoring the UV-Vis spectra of the V^{3+} electrolyte including Pt/C stored at 60 °C under atmosphere. As seen in **Supplementary Figure 5**, the peak for V^{4+} (760 nm) increased with the storage time, which reveals V^{3+} is oxidized to V^{4+}, coupled with Pt/C-catalysed oxygen reduction reaction. The result emphasizes the need of inert atmosphere and removal of the residual oxygen from the reactant solution.

Supplementary Note 1 Calculation of states of charge (SOC)

SOC of initially charged electrolyte was calculated using below equation[2,3].

$$\text{Positive half-cell: } E^{+} = \mathrm{E}^{\circ+\prime} - \frac{\mathrm{RT}}{\mathrm{F}}(\ln[\frac{1-SOC}{SOC}]) \quad \text{(S1)}$$

$$\text{Negative half-cell: } E^{-} = \mathrm{E}^{\circ-\prime} - \frac{\mathrm{RT}}{\mathrm{F}}(\ln[\frac{SOC}{1-SOC}]) \quad \text{(S2)}$$

$$\text{Full cell: } \Delta\mathrm{E} = \Delta\mathrm{E}^{\circ\prime} - \frac{2\mathrm{RT}}{\mathrm{F}}(\ln[\frac{1-SOC}{SOC}]) \quad \text{(S3)}$$

In the previous work of Kazacos's group, formal potentials for vanadium electrolyte of 1.6 M vanadium and sulfate level in 4 M ~ 4.2 M were calculated; $\mathrm{E}^{\circ+\prime}$ = 1.182 V for positive half-cell and $\mathrm{E}^{\circ-\prime}$= - 0.207 V for negative half-cell, respectively. Since the composition of reactor electrolyte (1.55 M of vanadium ion + 4.17 M sulfate ion) is almost identical to the electrolyte in previous work, these values were used for calculating the SOC of the reactor electrolyte after initial charging step. By using **Eq. S3**, the SOC of initially charged electrolyte was about 99% which means the electrolyte was fully charged when it was charged up to 1.9 V. SOC calculated from charge capacity exhibited same value (99%) consolidating the fact that high SOC was achieved by charging the electrolyte to 1.9 V. SOC of electrolyte charged up to 1.65 V also showed high SOC (98%), verifying that 0.8 V ~ 1.65 V is wide enough voltage range to check hydrogen evolution reaction.

Supplementary Note 2 Cost analysis for $V^{3.5+}$ electrolyte production

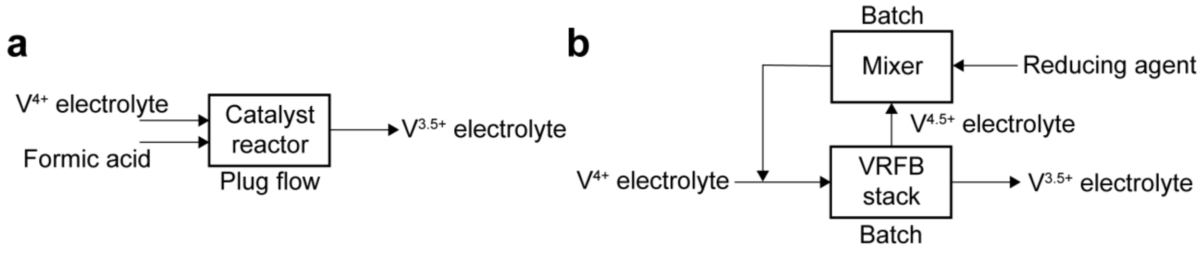

Supplementary Figure 6 Process diagrams for the production of 1.55 M $V^{3.5+}$ electrolyte

(a) Catalytic reaction, (b) conventional electrolysis method.

The process diagram of the newly proposed process for $V^{3.5+}$ electrolyte preparation using the catalytic reaction is given in **Supplementary Figure 6a**. Starting from V^{4+} electrolyte, the $V^{3.5+}$ electrolyte production can be achieved by a single step reaction using the catalytic reactor in a continuous manner. However, the conventional electrolysis method includes the external reduction step to fully utilize the feed vanadium solution (**Supplementary Figure 6b**), therefore, this recycle stream does result in the need for larger equipment and some additional processing cost.

Supplementary Table 1 Cost of the catalyst reactor (Production capacity: 40 L h^{-1})

Material		Specification	Unit	Unit cost ($)	Usage	Cost ($)
Reactor	Teflon body	¢ 300 ∗ ¢ 220 ∗ 300L	ea	625	2	1,250.0
	Teflon cap	¢ 300*105	ea	419.2	2	838.3
	1/4” male connector		ea	2.9	2	5.8
	SUS bolt	M8*55	ea		30	16.7
	Cost of labor					166.7
	Sub total					2,277.5
Pt/C-decorated carbon felt	Pt/C	TEC10E50 (Tanaka)	g	20.5[a)]	62	1,271.0
	Ionomer	D520 (DuPont)	L	316.7	0.72	228.0
	N-propyl alcohol		L	3.2	5.5	17.4
	Water		L	0.0033	4.5	0.015
	Carbon felt	GFD4.6 (SGL)	m^2	91.7	2.35	215.4
	Sub total					1,731.8
Total						4,009.3

a) Tanaka TEC10E50: 2460YEN/g

For a fair comparison with the large-scale electrolysis production using VRFB stack, catalyst reactor with production capacity of 40 L h^{-1} was considered. The large-scale reactor cost was analyzed as shown in **Supplementary Table 1**. The reactor mainly consists of a Teflon-based housing and Pt-decorated carbon felts. The catalytic activity of the reactor was measured to be $0.645\ L\ g_{Pt/C}^{-1} h^{-1}$, therefore, the required Pt/C catalyst for the reactor was 62 g (Pt: 28.8 g). Considering the amount of the catalyst and other components, the total cost of the reactor, which is capable of the production at 40 L h^{-1}, is $ 4,009 (US dollar). One can compare the stack cost at the same production capacity of 40 L h^{-1} to identify the capital cost difference.

Supplementary Table 2 Process cost for catalytic production of 1 L of 1.55 M $V^{3.5+}$ electrolyte production

Component	Requirement	Unit	Unit cost	Cost ($ L^{-1})
Formic acid	0.3875	mol L^{-1} [c)]	0.088 $ mol^{-1}	0.0341
Electrical heat input [a)]	55.14	Wh L^{-1}	0.15 $ kWh^{-1}	0.0083
Consumable [b)]	-	-	-	0.0072
Total				0.0496

a) Assumptions

1. Heat capacity of electrolyte: 3.7 kJ kg^{-1} $^{o}C^{-1}$
2. Heat recovery: 0.5 (fraction of total heat needed to raise temperature from 21 ^{o}C to 95 ^{o}C for reaction)
3. Electricity cost: 0.15$ kWh^{-1} (The same as that for the electrolysis)

b) Assumptions: Pt/C coated electrode need to be replaced every 6000 h operation.

c) L^{-1} in every unit refers to liter of vanadium solution.

Supplementary Table 3 Process cost for conventional method of 1 L of 1.55 M $V^{3.5+}$ electrolyte production

Component	Requirement	Unit	Unit cost	Cost ($ L^{-1})
Reducing agent [a)]	0.04	kg L^{-1} [c)]	1.2 $ kg^{-1}	0.0480
Electricity consumption	0.17	kWh L^{-1}	0.15 $ kWh^{-1}	0.0255
Consumable [b)]	-	-	-	0.0080
Total				0.0815

a) SO_2 was used as reducing agent.

b) Assumptions: Stack electrode need to be replaced every 500,000 L VRFB electrolyte preparation.

c) L^{-1} in every unit refers to liter of vanadium solution.

The process costs of the newly proposed catalytic production and conventional electrolysis were calculated as given in **Supplementary Table 2, 3**. For the catalytic production, the process cost for 1 L of $V^{3.5+}$ electrolyte is about $ 0.0496, while that for the electrolysis production is $ 0.0815. Therefore, 40% process cost reduction can be achieved with the catalytic production in comparison with the electrolysis due to the simplicity of the process. The calculation does not include labor cost, however, there will be a wider disparity between two processes when the labor cost is included. It should be noted that the other material costs (vanadium oxide and sulfuric acid) are identical between the two processes.

Supplementary References

1 Tang, L., Li, X., Cammarata, R. C., Friesen, C. & Sieradzki, K. Electrochemical stability of elemental metal nanoparticles. *J. Am. Chem. Soc.* **132**, 11722-11726 (2010).

2 Corcuera, S. & Skyllas-Kazacos, M. State-of-charge monitoring and electrolyte rebalancing methods for the vanadium redox flow battery. *European Chemical Bulletin* **1**, 511-519 (2012).

3 Buckley, D. N. *et al.* Method for determining the state of charge of a vanadium redox flow battery. Patent Application US 15/101,092 (2016).
