# Supplementary material for: Catalytic production of impurity-free V3.5+ electrolyte for vanadium redox flow batteries
Source: Nat Commun. 2019 Sep 27;10:4412. doi: 10.1038/s41467-019-12363-7 (PMC6764956; doi:10.1038/s41467-019-12363-7)
Supplement: Supplementary file 3 — Description of Additional Supplementary Files [file 41467_2019_12363_MOESM3_ESM.pdf]

Description of Supplementary Movie 1 file.

Supplementary Movie 1 shows continuous production of $V^{3.5+}$ electrolyte by using flow reactor with a production speed of 1 L h^{-1}. To reduce the play time and size of the file, video is played 500 times faster than its original speed.
